# Competitive Anxiety, and Guilt and Shame Proneness From Perspective Type D and Non-type D Football Players

**DOI:** 10.3389/fpsyg.2021.601812

**Published:** 2021-03-24

**Authors:** Adriana Kaplánová

**Affiliations:** Faculty of Physical Education and Sports, Comenius University, Bratislava, Slovakia

**Keywords:** worry, somatic anxiety, concentration, personality, sports psychology

## Abstract

The precompetitive, competitive, and postcompetitive mental states of athletes are currently not sufficiently researched. Long-term exposure to stressors contributes to the formation of mental blocks and leads to various health problems. One of the factors that can explain the variability of athletes' reactions to stress is their personality. This study is the first to examine competitive anxiety, and guilt and shame proneness in the context of the reaction of football players to distress in sports. The study consists of 112 male football players aged 16–24 (21.00 ± 1.90) who were divided into type D and non-type D football players according to scoring on the Denollet Scale (DS14). Football players also filled out the Sport Anxiety Scale (SAS-2) and the Guilt and Shame Proneness Scale (GASP). The taxonomic approach was conducted to test and to examine differences in stressor intensity as a function of type D personality. A correlation, multivariate analysis of variance, and regression analysis were performed in the study. We found that type D football players were more afraid of failures in sports (worry), felt more often autonomous excitement concentrated in the stomach and muscles (somatic anxiety), and showed more frequent concentration disruption than did non-type D football players. We also found that although type D football players were more likely to rate their behavior as negative and inappropriate, they showed a much greater effort to correct it than did non-type D football players. Negative affectivity and social inhibition of type D football players were positively correlated with competitive anxiety. In addition, we noted lower levels of somatic anxiety and lower concentration disruption for football players who used escape strategies to manage stress. The shame proneness subscale monitoring negative self-evaluation was also closely related to the concentration disruption of football players. We found that the examination of athletes by type D personality is necessary due to the fact that negative affectivity and social inhibition are significant predictors of competitive anxiety of football players, which explains their worries at 24.0%, somatic anxiety at 8.2%, and concentration disruption at 10.6%.

## Introduction

Stress is associated with the perception of threat, with resulting anxiety discomfort, emotional tension, and difficulty in adjustment (Selye, 1976). Athletes often feel tremendous pressure in competition, which is largely influenced by their level of aspiration (Kumar, 2013; Strandell, 2017; Buckley, 2018; Davis et al., 2019). According to Lewin's theory, after an experience with success, there is usually an increase in the aspiration level and, in the event of failure, decrease (Lewin, 1951; Buckley, 2018). If athletes do not approach their aspirations, there is a target discrepancy, which athletes experiencing frustration or deprivation (Kaplánová and Gregor, 2019b). The stressors that affect athletes can be divided into real, but also potential. While real stressors really threaten and disrupt the quality of life of athletes, potential stressors are related to the ability to imagine a stressor in such quality and intensity that they evoke the same physiological response as a real stressor (Steimer, 2002; Godoy et al., 2018; Kaplánová, 2020). This is related to the cognitive component of anxiety, which is responsible for fear and worries from poor sports performance, loss of popularity, or financial benefits, which have proven to be a significant factor in increasing the cognitive component of anxiety (Kaplánová, 2020). Worries and the fear of failure elicit a physiological response from the body to the fact that a stressor is present at a given moment. Irregular breathing causes stiffness in several muscle groups, often associated with increased respiratory rate, gastric tremors, excessive sweating, or excessive loss of saliva, for which the somatic component of anxiety is responsible (Martens et al., 1990; Heer et al., 2014; Jackson et al., 2017; Kaplánová, 2019b, 2020; Sun et al., 2020). In team sports, higher levels of cognitive and somatic anxiety are associated with disruption concentration. It turned out that more anxious athletes achieved lower peaking under pressure and lost their positive and enthusiastic attitude responsible for a bounce back from mistakes and failures. The higher level of the cognitive component of anxiety has made the coach's constructive criticism more hurtful, and football players took it too personally, which is also one of the reasons why attention is currently focused more on competitive anxiety and effective ways to reduce its level (Kaplánová, 2019a,c, 2020).

Guilt and shame are emotions that are negatively saturated and often associated with failure in sports (Lazarus, 2000; Mosewich et al., 2011; Hofseth et al., 2015). Guilt is not as painful and destructive as shame. This is due to the fact that the athlete's shame is intensely felt at the moment (lost match) but disappears over time. Guilt is rather an episodic emotion associated with repetitive thoughts of undesirable behavior (lost goal-scoring opportunity), which cause athletes unpleasant feelings and inner tension and evokes remorse (Tangney and Dearing, 2003; Tangney et al., 2007; Sheikh and Janoff-Bulman, 2010; Tangney and Tracey, 2013; Shen, 2018; Kaplánová and Gregor, 2019a,b; Julle-Danière et al., 2020; Kaplánová, 2020; Zhao et al., 2020). Guilt proneness is more related to self-observation, the study of internal negative experiences, mental processes, and states that take place in consciousness. During the introspection of negative experiences in sports, frequent mistakes occur, such as excessive selective focus attention on sports performance in which the athlete has failed in the past associated with the fear of recurrence of this experience. It is a loss of mental relaxation, which is often associated with the disintegration of movement stereotypes, deautomatization of game automatics, discoordination of movement, or general discontinuity (Kaplánová and Gregor, 2019b). When athletes feel shameful, they attribute it primarily to their personality and negative evaluations of themselves. The long-term effect of negative thoughts significantly reduces self-esteem and increases the fear of failure in sports (Kaplánová and Gregor, 2019b). Low self-esteem affects learning processes, overall adaptation, and coping with new situations, which is the reason why it is necessary to deal with this issue in sports (Fredrickson, 2013; Kaplánová, 2019b).

Fear is an immediate response to a particular impending stimulus. In sports, such an incentive can be an unexpected game situation resulting in a potential, light, or severe injury. Anxiety is a less intense but more lasting reaction that is associated with worries that the situation repeats itself (Steimer, 2002; Ford et al., 2017; Marshall et al., 2017; Zhang et al., 2018). Long-term experience of anxiety has an impact on the health of athletes. It is manifested by headaches, back pain, stiff neck and shoulders, insomnia, diarrhea, constipation, and palpitations. From an emotional point of view, it is associated with feelings of inferiority, futility, loneliness, irritability, apathy, and depression (Song et al., 2016; Kaplánová, 2019a, 2020). There are also intellectual deficits, which are manifested by a reduction in the ability to learn, think logically, and concentrate, as well as the ability to perform more complex tasks, the presence of which are barriers to successful sports performance (Schneiderman et al., 2005; Wu et al., 2020). A deeper study of the issue has contributed to the creation of a type D personality that describes the body's response to distress (Mols and Denollet, 2010; Kupper and Denollet, 2018; Spek et al., 2018). Type D personality is characterized by a high level of negative affectivity and a high level of social inhibition (Sher, 2005; Kasai et al., 2013; Li et al., 2016; Jandackova et al., 2017; Borkoles et al., 2018; Son et al., 2018). The synergy of both variables, according to Mols and Denollet (2010), is a risk factor with which various other health complications are associated. Negative affectivity expresses a person's tendency to experience negative emotions (Fruyt and Denollet, 2002; Conti et al., 2016). According to many experts, it is often associated with angry to anxious human reactions, as well as mood swings, frequent irritability stemming from internal nervousness, and deepening depression (Denollet and Conraads, 2011; Epifanio et al., 2018). Social inhibition, in turn, expresses a person's tendency to suppress expressed emotions and persistently hide from others (Condén, 2014; Dritto et al., 2015). It is closely linked to the fear of rejection, as well as one's tendency to perceive the world as threatening, leading not only to evasive reactions but also to isolation from others (Mols and Denollet, 2010; Vroege et al., 2019). According to Kaplánová and Gregor (2019b), athletes are already exposed to low, medium, or high load intensity in training, depending on whether aerobic activities with medium load intensity or anaerobically with high load intensity predominate. Disruption of the internal stability of the organism occurs at low to medium intensity, so it is possible to consider athletic youth as a population that faces stressors repeatedly and for a long time, which predisposes them to experience stress and negative emotions more often than in the general population (Gregor et al., 2019; Kaplánová, 2019b,c; Kaplánová and Gregor, 2019a,b).

In the current study, we focus on the body's response to distress in sports, which allows football players to be divided into type D and non-type D personalities. We examine their negative affectivity and social inhibition in the context of competitive anxiety and guilt and shame proneness in new contexts beneficial for the development of psychological training of football players. Currently, there are only a few studies dealing with type D personalities in the sports context (Borkoles et al., 2018). Also, only a few studies deal with competitive anxiety in individual and team sports (Verdaguer et al., 2016; Cho et al., 2019; Ponseti et al., 2019; González-Hernández et al., 2020; Reigal et al., 2020; Vila et al., 2020) and guilt and shame proneness of athletes, which plays a key role in creating mental blocks from a failed performance (Murrar et al., 2019). As we consider the examination of this connection to be justified, and currently there is no research of this type, we decided to formulate the following research questions in the presented study: (1) Are there statistically significant differences in competitive anxiety, and guilt and shame proneness between type D football players and non-type D? (2) Is there a statistically significant relationship between competitive anxiety, and guilt and shame proneness and type D football players? (3) Can negative affectivity and social inhibition be a statistically significant predictor of worry, somatic anxiety, and concentration disruption of football players?

## Methods

### Participants

The research involved 112 male football players aged 16–24 years (mean ± SD; 21.00 ± 1.90) with experience in their sports from 4 to 18 years (mean ± SD; 12.11 ± 2.93). At the time of filling in the psychological test batteries, all participants were active football players registered in the Slovak Football Association (SFZ). To classify subjects as type D or non-type D, we used the cutoff scores proposed by Denollet (2005). Football players who scored ≥10 in negative affectivity and social inhibition were assigned to a type D personality (*n* = 37). Those who scored ≤10 to non-type D personality (*n* = 75). The division of football players by type D personality was based on the recommendations of Borkoles et al. (2018), who applied and verified this procedure in sports.

### Measures

#### Sport Anxiety Scale

The Sport Anxiety Scale (SAS-2) was created to measure the competitive anxiety of athletes. It is composed of 15 items evaluating different aspects of anxiety, specifically, anticipatory anxiety (worry), anxious arousal during a task (somatic anxiety), and concentration disruption. Anticipatory anxiety involves concerns about performing poorly and the resulting negative consequences. Somatic anxiety involves various indices of autonomic arousal centered in the stomach and muscles. And the concentration disruption involves difficulties in focusing on task-relevant cues. The response format for each item consists of a linear 4-point scale ranging from 1 (no at all) to 4 (very much). The score for each subscale is calculated as the mean of the scores of subscale items, with a low score indicating a less intense form of that type of competitive anxiety and a high score indicating a high probability of exhibiting that type of anxiety (Smith et al., 2006). Cronbach's alpha based on the classical items analysis was 0.89 for worry, 0.79 for somatic anxiety, and 0.81 for concentration disruption.

### Guilt and Shame Proneness Scale

The Guilt and Shame Proneness Scale (GASP) consists of 16 items representing various forms of offenses that were supposed to evoke feelings of guilt or shame. Guilt proneness consists of subscales: negative behavior evaluation, which monitors the extent to which football players consider their behavior to be negative or unacceptable, and guilt repair, which represents football players' attempt to atone for behavior that they consider negative, inappropriate, or dishonest. Shame proneness consists of a subscale of negative self-evaluation that represents a negative relationship to oneself and shame withdraw, which monitors the football player's evasive reactions to the detection of a violation of social norms. Football players answered various scenarios, causing guilt and shame, on a 7-point scale from 1 (very unlikely) to 7 (very likely) (Cohen et al., 2011). In this study, Cronbach's alpha was 0.75 for negative behavior evaluation, 0.72 for guilt repair, 0.65 for negative self-evaluation, and 0.75 for shame withdraw, which testifies to the good internal consistency of the items of the scale.

#### Denollet Scale

The Denollet Scale (DS14) provides a taxonomic assessment of a person in distress by measuring signs of negative affectivity and social inhibition. It is a research diagnostic tool used to analyze the psychosocial determinants of human behavior. It consists of 14 statements that can be answered on a 5-point scale from 0 (false) to 4 (true). Type D personality is characterized by a high level of negative affectivity and a high level of social inhibition. Negative affectivity monitors the tendency of football players to experience negative emotions in borderline situations. Social inhibition, in turn, tends to suppress football players' emotions in social interactions and choose avoidant stress management strategies (Denollet, 2005). By calculating Cronbach's alpha, we verified the internal consistency of the items, which was 0.83 for negative affectivity and 0.86 for social inhibition.

### Procedure

SAS-2, GASP, and DS 14 were applied by a sports psychologist (author of the study) with years of experience in the field. The psychological test batteries were provided in a single booklet, which was given to football players. In all the cases, the psychological scales were filled anonymously, and participation in the study was entirely voluntary. The study design was approved by the Ethics Committee of Comenius University in Bratislava, Slovakia. All participants were informed about the aims, methods of data collection, and their use for research purposes. In the case of research participants under the age of 18, consent was provided by legal guardians. The data were collected in accordance with the provisions of the Declaration of Helsinki.

### Statistical Analysis

The data were analyzed by using SPSS (Version 23 for Windows; IBM, Armonk, NY, USA). The internal consistency items of SAS-2, GASP, and DS14 were tested by Cronbach's alpha, which showed acceptable values in the range of 0.65 to 0.89. Data were also checked by Kolmogorov–Smirnov and Shapiro–Wilk normality tests to allow appropriate statistical tests to be used for normal data distribution. Correlation analysis was performed using Pearson's correlation coefficient. Multivariate analysis of variance (MANOVA) simultaneously comparing means for multiple dependent variables across two or more groups was used. MANOVA main effects were further analyzed with Bonferroni-adjusted univariate ANOVA to test the differences between type D and non-type D football players. Regression analysis to examine the role of type D personality was conducted. Competitive anxiety and Guilt and Shame proneness were the dependent variables, and social inhibition and negative affectivity entered at step 1 and centered SI × NA at step 2 were the predictor variables.

## Results

A list of study variables with their possible score ranges, mean scores, standard deviations, and differences between type D and non-type D football players is presented in Table 1. A MANOVA with the two groups, type D personality and non-type D personality, as independent variables and competitive anxiety and guilt and shame proneness as dependent variables was carried out. MANOVA showed a significant result, F_(7,104)_ = 18.55, *p* = 0.01, η2 = 0.14. Type D football players scored significantly higher than did non-type D football players on worry, as well as on somatic anxiety, F_(7,104)_ = 3.87, *p* = 0.52, η2 = 0.03, and on concentration disruption, F_(7,104)_ = 4.22, *p* = 0.42, η2 = 0.04. There was also a statistically significant difference in guilt repair based on type D and non-type D personality of football players. Type D football players scored significantly higher than non-type D football players on guilt repair, F_(7,104)_ = 5.24, *p* = 0.24, η2 = 0.05. There were significant differences neither for type D and non-type D personality in negative behavior evaluation, F_(7,104)_ = 0.04, *p* > 0.05, η2 = 0.00, nor for negative self-evaluation, F_(7,104)_ = 0.77, *p* > 0.05, η2 = 0.01, and shame withdraw, F_(7,104)_ = 2.04, *p* > 0.05, η2 = 0.02.

**Table 1:** List of study variables with their possible score ranges, mean scores, standard deviations, and differences between type D and non-type D football players.

		**Football players (*****n*** **=** **112**)			
**Variables**	**Range**	**Type D** **(*n* = 37) M ± SD**	**Non-type D** **(*n* = 75) M ± SD**	***p***	**η2**
Worry	5–20	13.51 ± 3.48	10.20 ± 3.99	**0.000**	0.14
Somatic anxiety	5–20	10.22 ± 2.87	9.09 ± 2.83	**0.052**	0.03
Concentration disruption	5–20	8.95 ± 2.86	7.83 ± 2.64	**0.042**	0.04
Negative behavior evaluation	4–28	22.65 ± 3.64	22.83 ± 4.83	0.843	0.00
Guilt repair	4–28	14.11 ± 4.95	11.69 ± 5.39	**0.024**	0.05
Negative self-evaluation	4–28	18.84 ± 4.12	19.72 ± 5.38	0.382	0.01
Shame withdraw	4–28	19.57 ± 4.66	20.95 ± 4.88	0.156	0.02

*M, mean value; SD, standard deviation; p, significance; η2, eta squared. Statistically significant results are indicated in bold*.

Pearson's correlation analysis was used to test the relationships between the competitive anxiety, and guilt and shame proneness, social inhibition, and negative affectivity of football players. The correlation matrix of the variables is presented in Table 2. The results of the study showed a positive statistically significant relationship between the components of competitive anxiety and social inhibition of football players (r_worry_ = 0.35, *p* < 0.01; r_somaticanxiety_ = 0.19, *p* < 0.05 and r_concentrationdisruption_ = 0.21, *p* < 0.05), as well as negative affectivity of football players (r_worry_ = 0.48, *p* < 0.01; r_somaticanxiety_ = 0.32, *p* < 0.01 and r_concentrationdisruption_ = 0.34, *p* < 0.01), and a negative statistically significant relationship between somatic anxiety and shame withdraw (*r* = −0.25, *p* < 0.01), as well as concentration disruption and negative self-evaluation (*r* = −0.23, *p* < 0.05) and shame withdraw (*r* = −0.22, *p* < 0.05).

**Table 2:** Correlation matrix of the variables of competitive anxiety, and guilt and shame proneness, social inhibition, and negative affectivity.

**Variables**	**W**	**SA**	**CD**	**SI**	**NA**	**NBE**	**GR**	**NSE**	**SW**
Worry									
Somatic anxiety	0.55^**^								
Concentration disruption	0.58^**^	0.47^**^							
Social inhibition	0.35^**^	0.19^*^	0.21^*^						
Negative affectivity	0.48^**^	0.32^**^	0.34^**^	0.34^**^					
Negative behavior evaluation	0.14	−0.05	−0.10	0.02	−0.06				
Guilt repair	0.06	0.08	0.14	0.12	0.15	0.14			
Negative self-evaluation	−0.07	−0.13	−0.23^*^	−0.12	−0.11	0.45^**^	0.10		
Shame withdraw	−0.16	−0.25^**^	−0.22^*^	−0.05	−0.15	0.41^**^	−0.07	0.61^**^	

** p ≤ 0.01,

* *p ≤ 0.05*.

The results of the regression analysis for type D football players are presented in Table 3. Regression analysis showed negative affectivity as a statistically significant predictor of competitive anxiety of football players, which together with social inhibition explained worry 24.0%, somatic anxiety 8.2%, and concentration disruption of football players 10.6% of the variances. Type D personality was a statistically significant predictor for all components of the competitive anxiety of football players. Regression analysis where guilt and shame proneness (dependent variables) and social inhibition and negative affectivity of football players (independent variables) was performed. The predictors were not detected.

**Table 3:** Results of the regression analysis for type D football players.

	**Step 1** **R2 (%); beta predictors**	**Step 2** **R2 (%); beta predictors**
Worry	26.8%; SI = 0.208^*^, NA = 0.409^**^	24.0%; SI × NA = 0.497^**^
Somatic anxiety	10.7%; SI = 0.092, NA = 0.284^**^	8.2%; SI × NA = 0.300^**^
Concentration disruption	12.3%; SI = 0.107, NA = 0.300^**^	10.6%; SI × NA = 0.326^**^

*SI, social inhibition; NA, negative affectivity*.

** p ≤ 0.01,

* *p ≤ 0.05*.

## Discussion

A competition is a natural culmination of the training of athletes, during which they make all available physical and mental efforts to fight for the best possible result. The sports competition places high demands on the participants of the competition, which makes it stressful for athletes (Gregor, 2013; Kaplánová, 2019a,b, 2020; Kaplánová and Gregor, 2019b). The precompetitive, competitive, and postcompetitive mental states of athletes are currently not thoroughly examined, as evidenced by the fact that the present study is the first study on competitive anxiety, and guilt and shame proneness from the perspective of a type D personality monitoring the body's response to distress in sports. The usefulness of examining athletes according to personality type D was also pointed out by Borkoles et al. (2018), who also noted the occurrence of increased negative affectivity and social inhibition in the research group of athletes.

The present study provides evidence that the competitive anxiety of football players also varies depending on the Denollet personality type. Football players who announced attention problems before and during the competition experienced fear and worries of poor performance and felt autonomous excitement concentrated in the stomach and muscles belonged to the group of football players type D. Personality type D describes the body's response to distress by monitoring the level of their negative affectivity and social inhibition (Denollet, 2005; Mols and Denollet, 2010). Higher scores in these dimensions indicate an increased incidence of negative emotions of athletes, as well as the choice of avoidant stress management strategies. Type D personality was a statistically significant predictor for all components of the competitive anxiety of football players. According to research by several experts, the synergy of both variables poses an increased risk of health complications associated with physical and mental discomfort, which can culminate in heart problems and diabetes or later develop into anxiety and depressive mental disorders (Mols and Denollet, 2010; Kang et al., 2015). The study by Kaplánová (2020) suggests that one of the factors that increase the cognitive component of football players' anxiety is the fear of losing financial awards, prestige, or popularity, as well as the fear of a football club terminating the contract. In view of the above, we believe that by implementing continuous psychological training of football players aimed at reducing cognitive and somatic anxiety, it is possible to prevent the consequences of long-term stress. We recommend to athletes to adopt adequate relaxation techniques such as Schultz's autogenic training or Jacobson's progressive method, through which it is possible to teach the athlete's body to actively relax. Long-term benefits include lowering saliva cortisol levels, lowering heart rate and blood pressure, eliminating headaches, and increasing the overall quality of life (Wilk and Turkoski, 2001; Sheu et al., 2003; Anderson and Seniscal, 2006; Ghafari et al., 2009). Athletes with higher scores in competitive anxiety are advised to try a systematic desensitization technique that effectively reduces anxiety and helps overcome fear and inner turmoil. Systematic desensitization uses relaxation and imagination to remove a person's fear, worry, and anxiety from a certain object. At present, a direct confrontation with a real object is also used, which works on the principle of gradual steps and takes place in a certain sequence. The person is confronted with phobic situations until the fear of the object ceases to be so intense or disappears completely (Wolpe, 1961). The results of our research also showed that in the guilt repair dimension, type D football players scored higher than non-type D. We consider these results to be positive, as they predispose type D persons to correct behaviors that they evaluate as inappropriate or unacceptable, which can be a good starting point for voluntary and active participation in reducing anxiety or removing mental blocks.

The correlation matrix of variables competitive anxiety, guilt and shame, social inhibition, and negative affectivity of football players suggests several connections. The higher level of anticipatory anxiety of football players, which represents the cognitive component of anxiety, was closely related to higher negative affectivity and social inhibition. Worry about the failure of sports, which is usually part of athletes' precompetitive, competitive, and postcompetitive situations, seems to be closely linked to the creation of negative thoughts, as well as to the way athletes try to get out of the situation (Kaplánová, 2019a,c, 2020). In addition, we found that presumed anxiety is closely related to somatic anxiety as well as the duration of attention. The higher the level of predictable anxiety, the more football players experienced more frequent autonomic arousal concentrated in the stomach and muscles and felt disturbed concentration. Both variables also correlated with negative affectivity and social inhibition, which predispose type D football players to somatic difficulties as well as more frequent loss of concentration in performing tasks. According to Kaplánová and Gregor (2019b), athletes often resort to cognitive errors, such as inappropriate generalization, which is characterized by the evaluation of their performance on the basis of a single error that does not indicate their overall athletic performance. This cognitive error usually occurs immediately after the error has been made and is characterized by the angry or regretting reactions of athletes to the current failure in sports (Norcross and Prochaska, 1999; Kaplánová and Gregor, 2019b). Previous experience in sports also contributes to the creation of cognitive errors of athletes, such as excessive selective focus attention on situations in which athletes have failed. These are episodic concerns, which are often associated with the disintegration of movement stereotypes and the inability to focus attention or perform more complex tasks (Gregor, 2013; Kaplánová and Gregor, 2019b). If the minds of athletes get negative intrusive thoughts about a possible failure in sports, their chance to react optimally to various game situations also decreases. For this reason, it is necessary to eliminate cognitive errors in making judgments, e.g., through the cognitive technique of reframing (Kaplánová and Gregor, 2019b; Kaplánová, 2020). By reframing, sports psychologists can transform the negative contents in the athlete's mind into a more positive form that does not hinder sports performance (Hope et al., 2010; Mattila, 2015). The cognitive reframing technique allows athletes to look at the situation from a different perspective, helps them see the context in a broader context, and allows them to recognize and appreciate a positive view even in a seemingly negative situation (Vernooij-Dassen et al., 2011; Fredrickson, 2013).

The results of our research have also shown that the concentration disruption of football players is related to shame proneness. Football players who found themselves in a situation where they were ashamed had a higher negative self-evaluation and a higher tendency to choose escape reactions, but their concentration of attention was less disturbed than in the case of football players who tried to face situations with shame. Similar conclusions were reached by Nicholls and Polman (2007), who found that blocking negative thoughts and mentally withdrawing from a stressor can be an effective stress management strategy for elite athletes. Shame causes an unpleasant internal state, which leads to a disorganization of the structure of the self, which can last for varying lengths, depending on how much it is affected or damaged (Wheeler, 1995). From a physiological point of view, changes in heart rate or respiratory rate are present in shame, often externally demonstrated by reddening of the facial skin (Tangney and Tracey, 2013; Stuewig et al., 2015), which is consistent with the findings in our study. According to Kaufman (1989), denial of shame is manifested by defensive and compensatory mechanisms of human behavior, such as anger and excessive criticism, directed against oneself or others. Although denial may neutralize the apparent threat, there is a risk that its effect will be only short-lived (Nakonečný, 2013). Long-term and intense survival of shame carries the risk of excessive vulnerability or reactivity, which leads to violations of social norms or increases the risk of dishonest, immoral, and incorrect behavior toward others (Orth et al., 2010).

Human behavior is not just automatic response to an event but a complex and planned cognitive activity, reinforced by the so-called effect of strengthening, which may be based on a feeling of shame (Nakonečný, 2013), which is also one of the reasons why we believe that it is necessary to deal with shame proneness.

The presented study deals with personality of football players in the context of their reaction to distress in sports, which proves to be important especially from the aspect of maintaining the physical and mental health of athletes. As this is a pilot study that can contribute to the development of psychological training of football players, we recommend expanding knowledge in this area in other sports disciplines, or take into account the gender differences in precompetitive, competitive, and postcompetitive mental states between athletes. We consider the exclusion of gender differences of football players to be one of the limits of the presented study. We also base our assumptions on the study of Kristjánsdóttir et al. (2019), which points out the importance of examining psychological skills and anxiety in female football players. For this reason, we recommend that future research also focus on gender differences in competitive anxiety, and guilt and shame proneness in the context of the Denollet personality in female football players and take into account possible differences in the psychological training of female athletes.

## Conclusions

This study provides evidence that precompetitive, competitive, and postcompetitive conditions of athletes are closely linked to their personality and can also lead to serious physical and mental difficulties. The long-term effects of stress in sports, together with ineffective strategies for managing it, point to the need to develop psychological training for football players. The psychological training of football players should, therefore, be more focused on the practice of controlled muscle relaxation, the acquisition of breathing exercises, or the use of reframing techniques, which are much more effective and durable in reducing stress than avoidant stress management strategies. The results of our study also point to the need to train coaches or physical education teachers in the comprehensive preparation of athletes for the competitive conditions of sports.

## Data Availability Statement

The original contributions presented in the study are included in the article/supplementary material, further inquiries can be directed to the corresponding author/s.

## Ethics Statement

The studies involving human participants were reviewed and approved by Ethics Committee of Comenius University in Bratislava, Slovakia. Written informed consent to participate in this study was provided by the participants, or where necessary, the participants' legal guardian/next of kin.

## Author Contributions

AK contributed conception and design of the study, organized the database, performed the statistical analysis, and wrote the first draft of the manuscript.

## Conflict of Interest

The author declares that the research was conducted in the absence of any commercial or financial relationships that could be construed as a potential conflict of interest.
